# Web-Based Interventions to Improve Mental Health in Home Caregivers of People With Dementia: Meta-Analysis

**DOI:** 10.2196/13415

**Published:** 2019-05-06

**Authors:** Yinan Zhao, Hui Feng, Mingyue Hu, Hengyu Hu, Hui Li, Hongting Ning, Huijing Chen, Lulu Liao, Linlin Peng

**Affiliations:** 1 Xiangya School of Nursing Central South University Changsha China; 2 Xiangya-Oceanwide Health Management Research Institute Central South University Changsha China; 3 Department of Geriatrics Xiangya Hospital Changsha China; 4 National Clinical Research Center for Geriatric Disorders Xiangya Hospital Central South University Changsha China

**Keywords:** internet, education, mental health, caregivers, dementia

## Abstract

**Background:**

Dementia is a major cause of disability and dependency in older adults worldwide. It is often accompanied by general psychological distress, such as depression and anxiety symptoms, among caregivers of people with dementia (PwD). The physical and mental health of the caregiver is a prerequisite and a promise to help PwD continue to live as long and as well as possible. Web-based interventions can provide convenient and efficient support and an education tool to potentially reduce the negative outcomes associated with providing care.

**Objective:**

The aim of this study was to examine the effect of internet-based interventions on the mental health outcomes of family caregivers of PwD and to explore which components of the Web-based interventions play an important role.

**Methods:**

A comprehensive literature search was conducted in PubMed, Excerpta Medica dataBASE, PsycINFO, Cochrane Database, and the Cumulative Index to Nursing and Allied Health Literature using relevant terms such as Web-based and caregiver as keywords, covering all studies published before June 2018. A total of 2 reviewers independently reviewed all published abstracts, according to established inclusion and exclusion criteria. We extracted information about the participants, interventions, and results and reviewed article quality in terms of the randomized trial methods, using the approach recommended by the Cochrane Handbook for Systematic Reviews of Interventions.

**Results:**

A total of 815 caregivers participated in 6 studies, with 4 of the studies using depression as an outcome. The analysis found that depression scores dropped an average of 0.23 (95% CI −0.38 to −0.07; *P*<.01) after Web-based interventions. In 2 studies of caregivers who were experiencing anxiety symptoms, the average score for anxiety dropped by 0.32 points (95% CI −0.50 to −0.14; *P*<.01). However, in terms of coping, pain, and stress, the Web-based interventions showed a poor effect. On the whole, the addition of professional psychological support on the basis of education can improve caregivers’ mental health.

**Conclusions:**

Internet-based interventions were generally effective at reducing anxiety and depression in dementia caregivers, although negative results were found in some studies. As for burden and stress, further research is required.

## Introduction

### Background

Dementia is a syndrome, usually of a chronic or progressive nature, caused by a variety of brain illnesses that affect memory, thinking, behavior, and ability to perform everyday activities [1]. It appears that at least 2 cognitive changes result in significant social and occupational restriction [2]. Dementia currently affects approximately 50 million people worldwide, a number that is projected to grow to 82 million by 2030 and 152 million by 2050 [3,4]. With this growth, dementia is predicted to become the single greatest challenge facing health care and medical systems worldwide [5]. It is the second highest cause of disability in individuals aged 70 years and older and the seventh leading cause of death [4,6]. In terms of care costs, the total cost of dementia globally was US $1 trillion in 2018. By 2030, it is expected that the number will rise to US $2 trillion [7].

Dementia is one of the major causes of disability and dependency among the elderly worldwide [8]. Alzheimer’s Disease International (ADI) estimates that, globally, approximately 84% of elderly dementia patients live at home [9] and are cared for by nonprofessional family caregivers. Nonprofessional caregivers (NCs) assume responsibility for providing care in a nonprofessional, unpaid manner, based on family or affective ties [10]. However, because of a lack of knowledge and understanding of dementia, NCs do not understand why these elderly behave as they do or what the future may hold for them. In recent years, multiple studies have shown that, in caregivers of people with dementia (PwD), poor mental health is widespread, with anxiety and depression being the most common symptoms, reported by more than 50% of caregivers [11,12]. Mental health is a state of complete happiness, which refers to our ability to enjoy life and cope with challenges [13]. ADI reported higher levels of depression, emotional distress, and physical strain in caregivers of PwD than in caregivers of older adults with physical impairments [7]. Owing to the severity of this mental health situation, recent research also suggests that caregivers may be a high-risk group for suicide [14]. In Joling’s study, researchers found that over the course of 2 years, a number of people caring for a relative with dementia repeatedly considered hurting themselves, felt suicidal, or wished they were dead [15].

Providing training and support for the Alzheimer’s family and other family caregivers is an important public health priority. The World Health Organization (WHO) points out that we need to make a special effort to ensure that caregivers can obtain the information needed to do their work or training [16]. It is recognized that training programs for family caregivers of PwD can bring about positive results for caregivers [17], and researchers in this field are particularly interested in the training program methods that have been implemented. Ducharme et al [18] used keynote speeches to train family caregivers; increase their knowledge, practical skills, and caring skills; and improve their sense of self-efficacy. In a previous study, Hepburn et al [19] provided problem-solving skills and coping strategies for caregivers by issuing books and manuals to help alleviate caregivers’ anxiety and depression symptoms.

### Objectives

In recent years, because of the continuous development of the internet, internet-based intervention measures have gradually been applied by researchers, with Web-based interventions providing a more convenient and efficient support and education method for home care providers, as they allow caregivers to learn anytime, anywhere. Eysenbach [20] revealed that the efficiency with which medical services are delivered through internet interventions could reduce health care costs. In addition, particularly in remote and rural areas, caregivers may have easier access to the internet and electronic health care than to health care resources in the real world, thereby increasing equity in access to health care. The WHO also encourages primary care providers around the world to participate in e-learning training courses to improve their knowledge, skills, and confidence. Increased internet or mobile phone usage has opened up encouraging and novel outlets for mental health care dissemination and delivery efforts [21].

Currently, there are many systematic reviews of education and support for home caregivers based on the internet. Most focus on chronic disease caregivers and have shown positive results in terms of mitigating caregivers’ psychological issues [17,22-25]. Furthermore, these systematic reviews have summarized various education support measures and investigated their influence on the results of intervention measures for all kinds of care, such as mental health (anxiety, depression, and stress), general nursing results (self-efficacy, emergency coping styles, and social support), general health outcomes (quality of life [QoL], health, and satisfaction), and so on. Comprehensive results show that a Web-based intervention has resulted in positive, ineffective, or negative effects for caregivers. Overall, the results have been mixed, mainly because of methodological limitations, such as too few samples and too high dropout rates. During the search, we found there were only narrative reviews [26,27], and few meta-analyses were conducted on the mental health of caregivers of PwD in this field.

However, we believe that for different diseases, caregivers will have a different mental burden [28,29] and the focus and frequency of interventions will vary. Therefore, we designed and conducted a meta-analysis based on network intervention support for NCs of PwD, which is an extension of systematic evaluation and can be used to analyze comprehensive data using standardized statistical techniques [30]. We strictly evaluated the quality of the articles and critically evaluated the heterogeneity of the results, so that public health agencies can clearly understand current effective interventions in mental health training for Alzheimer’s care. The next step is to determine what measures should be taken to achieve widespread implementation of training.

## Methods

We conducted this systematic review and meta-analysis by following the Preferred Reporting Items for Systematic Review and Meta-Analysis guidelines [31].

### Study Design

A systematic review design was conceptualized using both data synthesis and descriptive analysis of randomized controlled trials (RCTs).

### Data Source and Retrieval Strategy

A comprehensive literature search was conducted in PubMed, Excerpta Medica dataBASE, Ovid MEDLINE, and Cochrane databases, covering all studies published before June 2018. We used the Cochrane systematic review method, internet-based terms related to care providers of the elderly and mental health as keywords. The retrieval strategy included correlative subject headings, keywords, and free words. A total of 2 reviewers developed search strategies in advance. Multimedia Appendix 1 lists the search terms of the system evaluation and the search strategies and search results of each database.

The 2 reviewers independently reviewed all published abstracts; extracted data on participants, interventions, and outcomes; and used the Cochrane risk of bias framework [32] to review article quality.

### Inclusion and Exclusion Criteria

To achieve high levels of evidence, we only chose RCTs for peer review. In addition, the inclusion criteria were as follows: (1) family caregivers of older adults with Alzheimer’s disease or other related types of dementia; (2) using the technology of the internet to provide intervention or support to the caregiver; (3) interventions aimed at improving the mental health of caregivers; and (4) RCTs with or without a blind method published in an English-language peer-reviewed journal. The exclusion criteria included the following: (1) a lack of real internet-based or browser-based intervention, such as video phone, or only adopting a non-internet–based video conference technology intervention; (2) lack of measurements of the outcome indicators of mental health (such as depression and anxiety symptoms) of caregivers; and (3) only providing Web-based education, without any interaction (such as discussion, Bulletin Board System, and feedback on problems).

The studies included a comparison group, defined as receiving no internet-based intervention, which could include blank controls, general controls, the use of paper materials, minimal guidance, or electronic communications for information resources (eg, e-bulletins).

### Quality Assessment

We used the Cochrane bias risk assessment tool [33] to assess the risk of bias of the included studies, which included the following 7 items: (1) generation of random sequences (selection bias); (2) allocation concealment (selection bias); (3) blinding of participants and personnel (performance bias); (4) blinding of outcome assessment (detection bias); (5) incomplete outcome data (attrition bias); (6) selective outcome reporting (reporting bias); and (7) other biases. A total of 2 reviewers independently rated the risk of bias in the above categories as *low risk*, *high risk*, and *unclear*.

### Data Extraction and Analysis

In total, 2 independent reviewers extracted and tabulated the following data for subsequent analysis: (1) participants (number of participants in the intervention and control groups, average age, and gender); (2) the intervention description (intervention, control group, and study duration); and (3) measurement of outcomes and measurement time. In the process of data extraction, some articles did not have a clear description of an intervention, and the duration of the intervention is sometimes difficult to determine. The problem was identified by contacting the corresponding authors of the published data.

In this study, we used standard mean difference (SMD) and 95% CIs to show the summary result, in which case it is necessary to mark the results of the study as a unified measure unit. For each study result, the difference between baseline, final value, and SD value was used for analysis. The analysis based on baseline change would be more effective and powerful than the comparison based on final value [34], as it removes a component of person variability from the analysis. In articles that did not report SD, we calculated SD from the reported mean, SE, 95% CI, and other information [35].

I^2^ was used to represent statistical heterogeneity of combinatorial studies [36]. I^2^ was used for quantitative statistics of the heterogeneity between the included studies. Under 30% indicated low heterogeneity, 30% to 60% moderate heterogeneity, and greater than 60% high heterogeneity. Review Manager (RevMan 5.3 version, Cochrane Library) was used for all analyses.

## Results

### Study Selection

**Figure 1:**
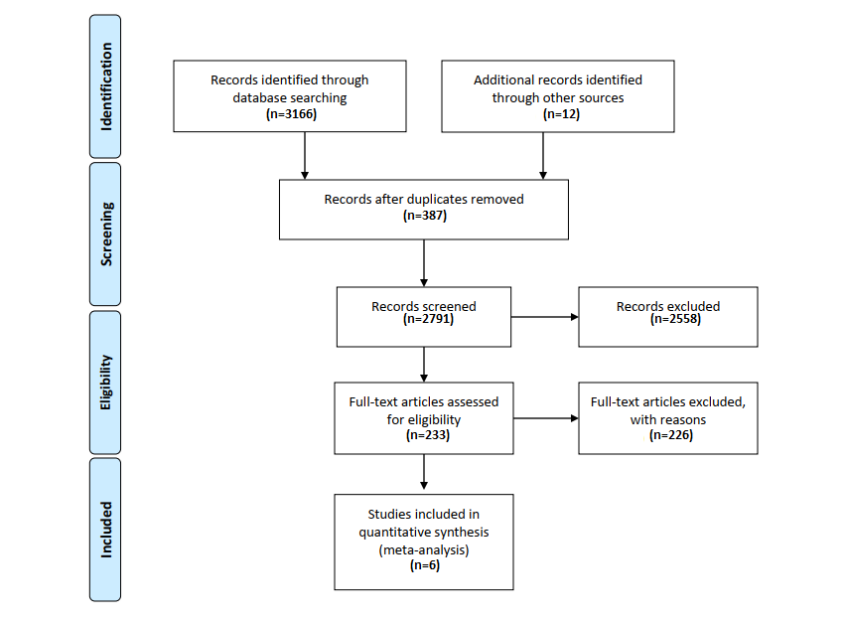
Flow diagram.

A total of 3166 studies were retrieved from various databases, with another 12 from other sources. Of the 3166 studies, 387 reported that the same study was excluded and 2558 did not meet the inclusion criteria. Next, a full-text review of the remaining 233 studies was conducted, including 17 systematic evaluations, 26 non-RCT studies, 73 intervention non-Web–based studies, 6 noncontrol articles, and 3 articles mainly targeted at professional caregivers. Finally, 6 articles were included in this study (Figure 1).

### Participants

A total of 815 caregivers of PwD or potential memory loss were included in the study. The mean age of the participants in the 6 studies in the report was 58.66 years. A total of 4 articles reported participant gender, with 73.5% of participants who were female. The participants, intervention, comparison, outcome measures, and results details of the included studies are shown in Multimedia Appendix 2.

### Types of Intervention

With the classification of Sherifali [25], we divided Web-based interventions for caregivers into single-component interventions and multicomponent interventions.

On the basis of the results of the literature retrieval, we found 2 single-component interventions using information or education and 4 multicomponent interventions, including information or education and peer support (3/4) and information or professional education and psychological support (1/4).

### Outcome Measures

According to Isabelle Dor’s [13] classification of mental health, the components of mental health include emotional well-being/QoL and psychological and social well-being. We used depression (4/6), anxiety (2/6), and stress/distress (3/6) as primary outcomes and coping (2/6), burden (4/6), and QoL (3/6) as secondary outcomes. The results of the analysis of each variable are as follows.

#### Depression

**Figure 2:**
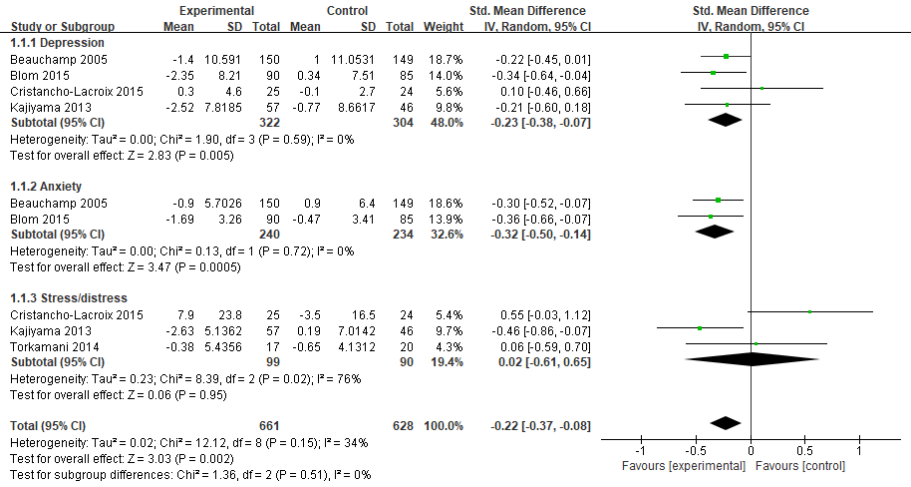
Forest plot for Web-based intervention on mental health. Std: standard.

**Figure 3:**
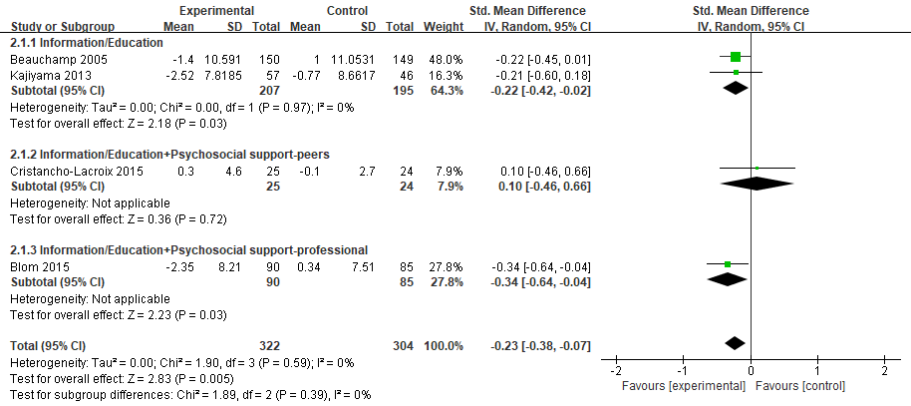
Forest plot for Web-based intervention on depression. Std: standard.

In the 4 studies [37-40], depression was used as the outcome variable, and Center for Epidemiologic Studies Depression Scale (n=3) and Beck Depression Inventory-II scale (n=1) were used to measure depression status. The sample size ranged from 49 to 299. For a sample of 626 caregivers, the overall combined effect of Web-based interventions on depression was statistically significant at −0.23 SMD (95% CI −0.38 to −0.07; *P*=.005). I^2^ was equal to 0%, showing low heterogeneity (Figures 2 and 3).

#### Anxiety

**Figure 4:**
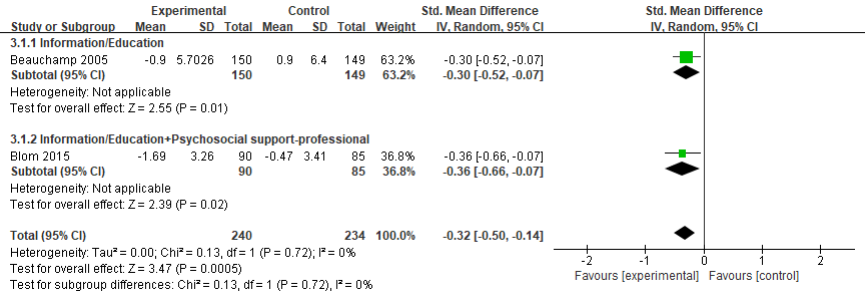
Forest plot for Web-based intervention on anxiety. Std: standard.

A total of 2 studies [37,38] included anxiety in the results, and State–Trait Anxiety Inventory (n=1) and Hospital Anxiety and Depression Scale (n=1) were used to measure caregiver anxiety status. The results showed that intervention in the experimental group significantly improved the anxiety status of caregivers with SMD of −0.32 (95% CI −0.50 to −0.14; *P*=.0005). I^2^ was equal to 0%, showing low heterogeneity (Figures 2 and 4).

#### Stress/Distress

In the 3 studies [39-41], we used caregiver stress as the outcome variable and used the Perceived Stress Scale (PSS; n=2). In another study, the researcher used the Neuropsychiatric Inventory (NPI) scale to sum up the frequency and severity scores of the caregivers’ problem behaviors to obtain the caregivers’ total distress score. The sample size ranged from 37 to 103 people. For a sample of 189 caregivers, the overall combined effect of the Web-based intervention on stress was not statistically significant (*P*>.05). I^2^ was equal to 32%, showing moderate heterogeneity.

#### Coping

Coping was used as the outcome variable in 2 studies [37,39]. One of the articles used the Revised Ways of Coping scale and the other used a self-made single item score to evaluate caregiver coping. For the sample of 209 caregivers, the overall combined effect corresponding to the network intervention method was not statistically significant (*P*>.05). I^2^ was equal to 0%, showing low heterogeneity.

#### Burden

Care burden was reported as an outcome in 3 studies [39,41,42]. Of them, 2 studies used the Zarit Burden Scale and the third set up an entry to evaluate care burden. For the sample of 184 caregivers, the overall combined effect corresponding to network intervention was not statistically significant (*P*>.05). I^2^ was equal to 0%, showing low heterogeneity.

#### Quality of Life

A total of 3 studies used QoL as outcome measures, using EuroQoL, QoL scale, and perceived QoL to assess changes in participants’ QoL. No statistical significance has been found for the intervention to improve caregiver QoL (*P*>.05). I^2^ was equal to 39%, showing moderate heterogeneity.

### Risk Bias

**Figure 5:**
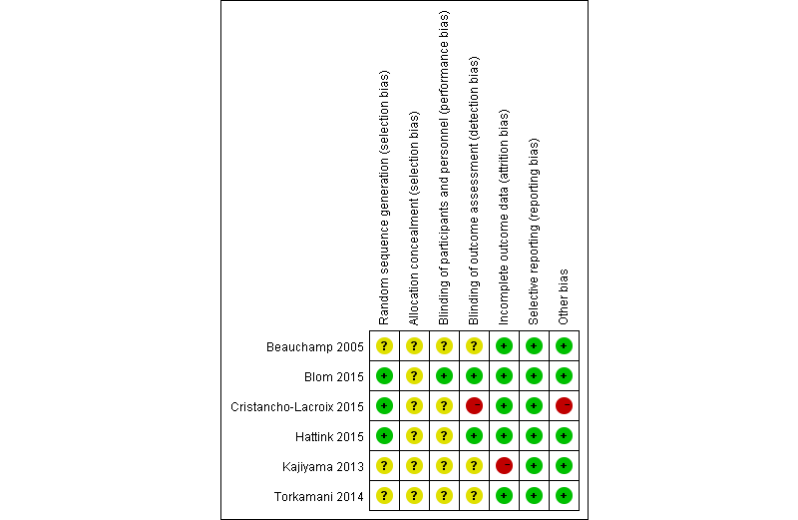
Risk of bias summary: review authors’ judgments about each risk of bias item for each included study.

The quality of the studies included was assessed by the Cochrane risk of bias summary. The overall risk of bias in the published literature is different, and owing to the lack of detailed descriptions reported in the literature, the risk of bias in many projects remains unclear. In all studies, the quality of performance bias and detection bias is relatively inconsistent, whereas the quality of publication bias is relatively low (Figure 5).

## Discussion

### Principal Findings

This is the first meta-analysis to only include RCT studies aimed at improving the mental health status of caregivers of older adults with dementia using a Web-based intervention. Many systematic reviews of the mental health of caregivers of people with chronic disease have previously been conducted [17,22-25]; however, the meta-analysis shows high heterogeneity owing to differences in study design, sample size, and intervention measures [25]. Our meta-analysis only included the RCT to constitute the most rigorous design and provide the highest quality evidence. We screened the samples strictly according to the inclusion and exclusion criteria and conducted a subgroup analysis to explore interventions that are the most beneficial for caregivers’ mental health, showing that the variable has low heterogeneity. However, the lack of research details and quality reports was a common problem in the published literature.

In this meta-analysis, although our analysis counted the aggregate effect sizes of different degrees of different categories of mental problems, in the 2 dimensions of anxiety and depression, the results were significant, which means that an internet-based intervention is indeed beneficial in improving anxiety and depression in caregivers of PwD. We calculated the effect size as Cohen's *d* [43] where positive effect size represents *symptom improvement* and negative effect size represents *symptom deterioration*. The depression (*d*=−0.21) and anxiety (*d*=−0.32) of caregivers in the support group showed low impact and medium impact, consistent with the previous results of Ling [44].

However, there has been no significant improvement in the stress, burden, coping, and life quality of the caregivers in the support group. In terms of burden and pain, only Kajiyama [40] and his colleagues revealed the effects of an intermediate (*d*=0.46) intervention. However, another study showed that stress symptoms decreased at 3 months but increased at 6 months. This may be caused by a small sample size, low test efficiency, high rate of loss to follow-up, and the evolution of the patient’s illness over time. A high rate of loss to follow-up is a common problem in psychotherapy research [45]. Moreover, owing to the lengthy and time-consuming nature of internet courses, the rate may be higher [46]. In the studies we included, the average follow-up rate loss was 28.3%, ranging from 0% to 41.5%. In addition, confusion about the stress source is another important reason, which Statistics Canada noted in 2009 [47]. Caregivers have multiple responsibilities in addition to their caregiver role, with 60% working at a paid job and 28% caring for children under the age of 18 years. These pressures are difficult to eliminate using a simple intervention. At the same time, in this review, we found that the heterogeneity of caregiver stress is high. Alba pointed out that continuous outcomes showed substantially higher I^2^ than meta-analyses of binary outcomes [48]. In this review, the source of high heterogeneity may be the difference in the evaluation tools of outcome. In their study, Cristancho-Lacroix [39] and Kajiyama [40] used a sophisticated stress assessment tool, the PSS; however, the caregiver stress data used by Torkamani came from the NPI.

All 6 studies used mature Web-based training platforms, such as STAR, iCare, and Diapason Program, but showed different results. We split the components, shown in Figures 3 and 4, and found that (1) for the depression and anxiety status of dementia caregivers, the effect of increasing professional support on the basis of information/education (SMD=−0.23, 95% CI −0.39 to −0.07) is better than the effect of increasing peer support (SMD=−0.16, 95% CI −0.34 to −0.02). Peeters et al [49] pointed out in a cross-sectional study of caregivers of 984 PwD in the Netherlands that most informal caregivers report they need additional information and advice, for example, about how to cope with their relative’s behavioral problems, about the illness trajectory progression, and emotional support and coordination of dementia care. Gaugler et al’s [50] empirical findings emphasize that the source of professional information can influence support recommendations for dementia caregiving families in need. On the contrary, in interviews conducted in 1 study [51], some caregivers who participated in peer support activities said that there were always 1 or 2 negative, unhappy people on the team who did not want others to be happy. In terms of pain, only information/education was given, which showed good results. Peer support and professional support were added, but the results were not statistically significant; (2) the improved and comprehensive stress management program showed better outcomes than the modified multicomponent integration program. In the research by Kajiyama [40] and Blom [38], a stress coping model was applied. The course mainly aims to teach nurses a set of core skills aimed at stress management, including relaxation training, learning to increase skills every day, cognitive restructuring, and asking peers/experts for advice. In other training platforms, information on Alzheimer’s disease, management of one’s emotions, and common processing problems are added on the basis of adjusting coping skills. Therefore, future studies should consider maintaining the systematic integrity of stress coping courses when revising the model; and (3) in his study, Beauchamp personalized the interventions based on the particular concerns of the viewer and the degree of dementia of the person receiving care. It showed good results for depression (*d*=−0.22), anxiety (*d*=−0.30), and stress (*d*=−0.39). This tailored intervention offers a promising and relatively inexpensive method of individualizing content to maximize relevance and impact.

According to the assessment of literature quality, all results of the examination were rated as low or medium, which may reflect the lack of consistency in this emerging field and the resulting research, such as inconsistency in theoretical model selection, inconsistency in intervention course content, and inconsistency in evaluation tools. In addition, the lack of information in some studies leads to an inability to assess the risk of bias in many areas, which is another reason for not showing consistent results. Although there is growing evidence that Web-based multicomponent interventions are feasible, there is no evidence as to which intervention components or platforms are the most useful and which could reduce the cost of training for NCs. Future research can be started from the following aspects: (1) pay attention to NCs of PwD in terms of providing psychological support; (2) when making revisions to the systematic course of stress and coping, pay attention to its integrity and systematisms; (3) for different types of caregivers, develop personalized intervention content that can be based on their needs; and (4) cost-effective interventions can be added in future research.

### Strengths and Limitations

This meta-analysis summarizes the effects of internet-based interventions that can be used to evaluate the mental health outcomes of home caregivers of older adults with dementia. Although there have been systematic evaluations in this field previously, we proposed rigorous screening of articles, only included RCTs, developed a comprehensive retrieval strategy, searched 5 databases to ensure we found the most comprehensive literature, and summarized the outcomes of different types of intervention studies by a subgroup analysis. However, after a comprehensive analysis, we found a number of limitations. In all of the studies that were included, the overall quality of evidence was poor, the sample size of some studies was insufficient, the intervention measures varied greatly, and the measurement tools of the outcome variables were different. This review also has some limitations. We divided Web-based interventions into 4 subgroups to reduce heterogeneity but, in some aspects, some heterogeneity remains.

### Conclusions

This is the first meta-analysis that only included RCT studies aimed at improving the mental health status of caregivers of older adults with dementia based on a Web-based intervention. Our research results show that in recent years, the literature on Web-based intervention measures has been emerging continuously. Although negative results were found in some studies, internet-based interventions were generally effective at decreasing anxiety and depression in caregivers. Next, the multicomponent internet interventions can be analyzed in further detail using a standardized assessment tool to analyze the outcome indicators to encourage researchers to adopt a more rigorous method in future.

Future researchers are encouraged to take a more rigorous approach and to continue to report on other mental health outcomes, such as stress and burden in dementia caregivers. Ongoing large-scale, high-quality studies are required.

## Appendix

Multimedia Appendix 1 Search terms.

Multimedia Appendix 2 Characteristics of included studies.

## Abbreviations

ADI: Alzheimer’s Disease International
NC: nonprofessional caregiver
NPI: Neuropsychiatric Inventory
PSS: Perceived Stress Scale
PwD: people with dementia
QoL: Quality of Life
RCT: randomized controlled trial
SMD: standard mean difference
WHO: World Health Organization
