# Supplementary material for: Web-Based Interventions to Improve Mental Health in Home Caregivers of People With Dementia: Meta-Analysis
Source: J Med Internet Res. 2019 May 6;21(5):e13415. doi: 10.2196/13415 (PMC6526687; doi:10.2196/13415)
Supplement: Multimedia Appendix 1 [file jmir_v21i5e13415_app1.pdf]

Mesh Terms		
(1) Internet；telemedicine；computer, handheld；cell phone; wireless technology<br>(2) Caregivers；family ；spouse; Home Nursing<br>(3) Education；health education; cognitive therapy		
**Key Words and Free Words**		
(1) Web；cyberspace ；cyber Space ；social media；distance learning；E-health ；Online ；on-line ；Skype ；webcam<br>(2) Family caregivers；home caregivers；<br>(3) intervention*；program*；“therap*；psycho-education*；psychoeducation*； training；support；Cognitive Therapy；psychological		
**1. PubMed**		
#1	Search(Internet [Mesh] OR telemedicine [Mesh] OR telecommunications [Mesh] OR (computers, handheld [Mesh]) OR wireless technology [Mesh] OR cell phone [Mesh])	6961
#2	Search(web[Title/Abstract] OR social media[Title/Abstract] OR blog*[Title/Abstract] OR social networking[Title/Abstract] OR twitter*[Title/Abstract] OR tweet*[Title/Abstract] OR online[Title/Abstract] OR instant* messag*[Title/Abstract] OR text messag*[Title/Abstract] OR text[Title/Abstract] OR texts[Title/Abstract] OR texting[Title/Abstract] OR facebook*[Title/Abstract] OR myspace[Title/Abstract] OR linkedin[Title/Abstract] OR email*[Title/Abstract] OR e-mail*[Title/Abstract] OR electronic mail[Title/Abstract] OR app[Title/Abstract] OR apps[Title/Abstract] OR message board*[Title/Abstract] OR electronic[Title/Abstract] OR e-health[Title/Abstract] OR telecommunication*[Title/Abstract])	25960
#3	#1 OR #2	31388
#4	Search(education[Mesh] OR health education[Mesh] OR cognitive therapy[Mesh])	9932
#5	Search(intervention[Title/Abstract] OR program[Title/Abstract] OR therap* [Title/Abstract] OR psycho-education[Title/Abstract] OR psychoeducation [Title/Abstract] OR training[Title/Abstract] OR support[Title/Abstract] OR psychological [Title/Abstract]	4116164
#6	#4 OR #5	85770
#7	Search(caregiver[Mesh] OR family[Mesh] OR Home Nursing[Mesh] OR spouse [Mesh])	3401
#8	Search(friend[Title/Abstract]OR acquaintance [Title/Abstract] OR adult child[Title/Abstract] OR carer*[Title/Abstract] OR care giver*[Title/Abstract] OR caregiver*[Title/Abstract] OR caretaker*[Title/Abstract])	2174
#9	#7 OR #8	4899
#10	Search(Dementia[Mesh])	2594
#11	Search (dementia*[Title/Abstract] or alzheimer*[Title/Abstract])	3946
#12	#10 OR #11	4240
#13	#3 AND #6 AND #9 AND #12	82

#14	Search ((((((((Randomized controlled trial[Publication Type]) OR Controlled clinical trial[Publication Type]) OR Randomized[Title/Abstract]) OR Ramdomized [Title/Abstract]) OR Randomised[Title/Abstract]) OR Ramdomised[Title/Abstract]) OR Randomly[Title/Abstract]) OR Trial[Title/Abstract]) OR Groups[Title/Abstract]	1911819 43
#15	#13 AND #14	
**2.Cochrane Database**		
#1	MeSH descriptor: [Internet] explode all trees	8466
#2	MeSH descriptor: [Telephone] explode all trees	15405
#3	MeSH descriptor: [Computer, handheld] explode all trees	334
#4	MeSH descriptor: [Wireless technology] explode all trees	229
#5	MeSH descriptor: [Cellphone] explode all trees	87
#6	(internet OR web OR cyberspace OR World Wide Web OR user-computer interfaceor mobile OR cellular phone OR social media OR smart-phone OR interactive OR Facebook OR twitter OR Tablet OR iPad OR distance learning OR e-health OR Online OR on-line oOR Skype OR webcam OR Videophone OR Telemedicine OR Wireless OR Wi-Fi):ti,ab,kw (Word variations have been searched)	92331
#7	#1 OR #2 OR #3 OR #4 OR #5 OR #6	105173
#8	MeSH descriptor: [Webcasts] this term only	65
#9	(webcast* or podcast* or streaming video* or RSS or really simple syndication or YouTube): ti,ab,kw(Word variations have been searched)	539
#10	#7 or #8 or #9	105597
#11	MeSH descriptor: [Caregivers] this term only	6992
#12	MeSH descriptor: [Family] this term only	29369
#13	MeSH descriptor: [Adult Children] this term only	33819
#14	MeSH descriptor: [Spouses] this term only	802
#15	MeSH descriptor: [Home Nursing] explode all trees	5735
#16	(carer* or caretaker* or care-giver* or caregiver*): ti, ab, kw(Word variations have been searched)	9757
#17	(adult child* or spous*): ti, ab, kw (Word variations have been searched)	34945
#18	#11 or #12 or #13 or #14 or #15 or #16 or #17	75199
#19	MeSH descriptor: [Dementia] explode all trees	18033
#20	(dementia* or alzheimer*): ti, ab, kw (Word variations have been searched)	14267
#21	#19 or #20	20337
#22	MeSH descriptor: [Education] explode all trees	58403
#23	MeSH descriptor: [Health education] explode all trees	33162
#24	MeSH descriptor: [Cognitive therapy] explode all trees	28433
#25	(intervention OR program OR training OR therp* OR psycho-education OR psychoeducation OR acquaintance): ti,ab,kw(Word variations have been searched)	266981

#26	#22 or #23 or #24 or #25	295120
#27	#7 and #10and #18and #21 and #26 content type: Trials	549
**3. CINAHL**		
S1	(MH "Internet")	24454
S2	(MH "computers handheld")	1305
S3	(MH "World Wide Web")	31989
S4	(MH "Social Media")	5354
S5	(MH "World Wide Web Applications")	2751
S6	(MH "Electronic Mail")	16613
S7	TI ((internet or web or social media or blog* or social networking or twitter* or tweet* or online or instant* messag* or text messag* or text or texts or texting or facebook* or myspace or linkedin or email* or email* or electronic mail or app or apps or message board* or electronic or ehealth or telecommunication*)) OR AB ((internet or web or social media or blog* or social networking or twitter* or tweet* or online or instant* messag* or text messag* or text or texts or texting or facebook* or myspace or linkedin or email* or email* or electronic mail or app or apps or message board* or electronic or ehealth or telecommunication*))	103533
S8	TI ((webcast* or podcast* or streaming video* or RSS or really simple syndication or youtube)) or AB ((webcast* or podcast* or streaming video* or RSS or really simple syndication or youtube))	1153
S9	(MH "Webcasts+")	382
S10	(MH "Social Networking")	1220
S11	S1 or S2 or S3 or S4 or S5 or S6 or S7 or S8 or S9 or S10	151606
S12	(MH "Caregivers")	15097
S13	TI ((carer* or caretaker* or caregiver* or caregiver*)) or AB ((carer* or caretaker* or caregiver* or caregiver*))	25888
S14	(MH "Family") or (MH "Adult Children")	20267
S15	(MH "Spouses")	4991
S16	TI ((adult child* or spous*)) or AB ((adult child* or spous*))	16935
S17	S12 or S13 or S14 or S15 or S16	64720
S18	(MH " Dementia ")	13906
S19	TI(dementia* or alzheimer*) or AB(dementia* or alzheimer*)	24907
S20	S18 or S19	28530
S21	(MH "Education")	5565
S22	(MH "Health education")	11259
S23	(MH "Cognitive therapy")	9835
S24	TI (intervention or program or therap* or psycho-education or psychoeducation or training or acquaintance) OR AB ((intervention or program or therap* or psycho-education or psychoeducation or training or acquaintance))	524721
S25	S21 or S22 or S23 or S24	538830
S26	S11 and S17 and S20 and S25	156

**4.PsycINFO**		
S1	(MH "Internet")	223
S2	(MH "computers handheld")	2
S3	(MH "World Wide Web")	169
S4	(MH "Social Media")	698
S5	(MH "World Wide Web Applications")	201
S6	(MH "Electronic Mail")	2155
S7	TI ((internet or web or social media or blog* or social networking or twitter* or tweet* or online or instant* messag* or text messag* or text or texts or texting or facebook* or myspace or linkedin or email* or email* or electronic mail or app or apps or message board* or electronic or ehealth or telecommunication*)) OR AB ((internet or web or social media or blog* or social networking or twitter* or tweet* or online or instant* messag* or text messag* or text or texts or texting or facebook* or myspace or linkedin or email* or email* or electronic mail or app or apps or message board* or electronic or ehealth or telecommunication*))	111731
S8	TI ((webcast* or podcast* or streaming video* or RSS or really simple syndication or youtube)) or AB ((webcast* or podcast* or streaming video* or RSS or really simple syndication or youtube))	912
S9	(MH "Webcasts+")	0
S10	(MH "Social Networking")	675
S11	S1 or S2 or S3 or S4 or S5 or S6 or S7 or S8 or S9 or S10	116092
S12	(MH "Caregivers")	3
S13	TI ((carer* or caretaker* or caregiver* or caregiver*)) or AB ((carer* or caretaker* or caregiver* or caregiver*))	24815
S14	(MH "Family") or (MH "Adult Children")	12
S15	(MH "Spouses")	12
S16	TI ((adult child* or spous*)) or AB ((adult child* or spous*))	29252
S17	S12 or S13 or S14 or S15 or S16	29264
S18	(MH " Dementia ")	1
S19	TI(dementia* or alzheimer*) or AB(dementia* or alzheimer*)	37075
S20	S18 or S19	37075
S21	(MH "Education")	5
S22	(MH "Health education")	1
S23	(MH "Cognitive therapy")	596
S24	TI (intervention or program or therap* or psycho-education or psychoeducation or training or acquaintance) OR AB ((intervention or program or therap* or psycho-education or psychoeducation or training or acquaintance))	448340
S25	S21 or S22 or S23 or S24	439096
S26	S11 and S17 and S20 and S25 Limiters-Language:English	10
**5. EMbase**		
#1	'internet'/exp OR internet	124809

#2	'internet’: ab,ti	58667
#3	'web':ab,ti	110304
#4	'social media'	14865
#5	'social media':ab,ti	9734
#6	'app':ab,ti OR 'apps':ab,ti	32164
#7	'twitter':ab,ti	2945
#8	'tweet*':ab,ti	1917
#9	'online':ab,ti	122704
#10	'message board*':ab,ti	221
#11	'instant* messag*':ab,ti	323
#12	text AND messaging	4341
#13	'text messag*':ab,ti	3941
#14	'text*':ab,ti	130903
#15	'facebook':ab,ti	3988
#16	'myspace':ab,ti	117
#17	'linkedin':ab,ti	213
#18	'e mail'	45065
#19	'email*':ab,ti	15397
#20	webcast	538
#21	'electronic':ab,ti	219859
#22	'social networking':ab,ti	2681
#23	'webcast*':ab,ti OR 'podcast*':ab,ti OR 'streaming video*':ab,ti OR 'rss':ab,ti OR 'really simple syndication':ab,ti OR 'youtube':ab,ti	5224
#24	#1 OR #2 OR #3 OR #4 OR #5 OR #6 OR #7 OR #8 OR #9 OR #10 OR #11 OR #12 OR #13 OR #14 OR #15 OR #16 OR #17 OR #18 OR #19 OR #20 OR #21 OR #22 OR #23	698510
#25	caregiver	80643
#26	'carer*':ab,ti OR 'care giver*':ab,ti OR 'caregiver*':ab,ti OR 'caretaker*':ab,ti	97006
#27	family	1251271
#28	adult AND child	740051
#29	spouse	20751
#30	'adult child*':ab,ti OR 'spous*':ab,ti	24456
#31	#25 OR #27 OR #28 OR #29 OR #30	198898
#32	dementia	195938
#33	'dementia*':ab,ti OR 'alzheimer*':ab,ti	261232
#34	#32 OR #33	300656
#35	education	1514781
#36	health AND education	745517
#37	cognitive AND therapy	172037
#38	'intervention':ab,ti OR 'program':ab,ti OR 'therap*':ab,ti OR 'psycho-education':ab,ti OR 'psychoeducation':ab,ti OR 'training':ab,ti OR 'support':ab,ti OR 'psychological':ab,ti	5827805

#39	#32 OR #33 OR #34 OR #35	1034082
#40	#24 AND #31 AND #34 AND #39	2429
#41	#24 AND #31 AND #34 AND #39 AND [english]/lim	2369
