# Supplementary material for: Web-Based Interventions to Improve Mental Health in Home Caregivers of People With Dementia: Meta-Analysis
Source: J Med Internet Res. 2019 May 6;21(5):e13415. doi: 10.2196/13415 (PMC6526687; doi:10.2196/13415)
Supplement: Multimedia Appendix 2 [file jmir_v21i5e13415_app2.pdf]

Study/Year	Participation (caregivers)	Intervention	Comparison	Outcome measures	Results
Beauchamp et al [37]/2005	150 caregivers of people with substantial memory problems in intervention group and 149 in control group	Caregiver's Friend: Dealing with Dementia<br><br>**key components:**①provides text material and videos for the caregivers' knowledge, cognitive, emotion, behavioral skill.<br><br>**Total duration:** 6 months access to intervention	Usual care and waitlist control.	There was not clear state primary outcomes, however there were four outcomes of interest:<br>(i)depression (CES-D);<br>(ii)state anxiety (the State-Trait Anxiety inventory);<br>(iii)caregivers gain (Positive Aspects of Caregiving survey);<br>(iv)caregiver strain (Caregiver Strain Instrument)	**Positive outcomes:** depression, anxiety, stress, caregiver strain and caregiver gain, self-efficacy, and intention to seek help.<br>**Negative outcomes:** ways of coping.
Kajiyama et al[40]/ 2013	57 caregivers of people with dementia in intervention group and 46 in control group	iCare stress Management e-Training program<br><br>**key components:** Eight topics about six modules and each module over a 7- to 10-day interval, embedded the video clips illustrating how to do skills presented.<br><br>**Total duration:** 3 months access to intervention	EOC：a website containing information about dementia and written materials	There was not clear state primary outcomes, however there were four outcomes of interest:<br>(i)perceived stress(PSS);<br>(ii)level of bother due to disruptive behaviors(RMBPC);<br>(iii)depression (CES-D);<br>(iv)Quality of life(PQoL)	**Positive outcomes：** perceived stress and level of bother due to disruptive in the ICC but not the EOC.<br>**Negative outcomes:** depressive symptoms and quality of life

Hattink et al[42]/2015	72 informal caregivers of person with dementia living in the community and 24 volunteers in community dementia cate.	STAR:(Skills Training and Reskilling)<br><br>**key components:**①provides online course with 8 modules on different topics in caregiving, provides text, video, interactive exercises(quizzes),knowledge tests, references to other website, literature videos.② tailored peer support (Facebook communication）<br><br>**Total duration:** 6 month access to intervention	Randomly allocated to a 4-mouth waitlist for access to STAR	**Primary:**(i)knowledge on dementia(ADKS);(ii)attitudes regarding dementia(2 questions)<br><br>**Secondary:**(i)empathy(IRI); (ii)quality of life(2 questions); (iii)burden(1 question); (iv)sense of competence(SSCQ)	**Positive outcomes：** empathy, knowledge, perspective and sense of competence. significant decrease in distress.<br><br>**Negative outcomes:** burden, attitude, quality of life.
Blom et al[38]/2015	245 family caregivers of people with dementia.	Mastery over Dementia (MoD)<br><br>**key components:** provides 8 lessons and booster session with the guidance of a coach monitoring progress and evaluating the homework. Each lesson consist of information (text material and videos).<br><br>**Total duration:**5-6 month access to intervention	Minimal intervention consisting of e-bulletins with practical information on caring for person with dementia. No contact with a coach.	**Primary:** depression (CES-D)<br>**Secondary:**<br>(i)symptoms of anxiety (HADS); (ii)perceptions of distress (Self-Perceived Pressure from Informal Care scale);	**Positive outcomes:** anxiety and depression<br><br>**Negative outcome**: distress

Cristancho-Lacroix et al[39]/ /2015	25 caregivers of people with dementia in intervention group and 24 in control group	Diapason<br><br>**key components:**①provides 12 weekly online sessions, each session include theoretical and practical information, videos of health professionals and practice guide for applying the session's content.②provides relaxation training, life story, the private and anonymous forum for caregivers to share with peers.<br><br>**Total duration:** 3 months access to intervention	Usual care.	**Primary:** stress of caregivers (PSS)<br>**Secondary:**<br>(i)self-efficacy (RSCS);<br>(ii)perception and reaction to cognitive or behavioral symptoms (RMBPC); (iii)subjective burden(Zarit Burden Interview);<br>(iv)depression symptoms(BDI-II);<br>(v)self-perceived health(NHP)	**Positive outcomes:** Knowledge and stress<br><br>**Negative outcomes:** burden, depression, reaction to problem behaviors, self-efficacy, self-perceived health, energy, coping and quality of relationship

Torkamani et al [41]/2014	60 caregivers of people with dementia from 3 site	ALADDIN<br><br>**key components:**①ALADDIN TV: information and education material, relaxation.②SOCIAL NETWORKING: forum for caregivers to communicate with each other.③MY TASKS: caregivers complete questionnaires about their own and relatives health that can generate clinical alerts, monitored by clinicians who can respond quickly;④CONTACT US: caregivers can alert clinical site and request contact<br><br>**Total duration:** 6 months access to intervention	Usual care	There was not clear state primary outcomes , however there were four outcomes of interest:<br>(i)burden(Zarit Burden Interview);<br>(ii)depression system(BDI and Zung);<br>(iii)distress (NPI);<br>(iv)Quality of life(EQ5D and Quality of Life Scale)	**Positive outcomes:** distress and quality of life<br><br>**Negative outcome:** burden and depression

Abbreviations: CES-D, Center for Epidemiologic Studies–Depression; PSS, Perceived Stress Scale; RMBPC, Revised Memory and Behavior Problems Checklist; PQoL, Perceived quality of life; ADKS, Alzheimer's Disease Knowledge Scale; IRI, Interpersonal Reactivity Index; SSCQ, Short Sense of Competence Questionnaire; HADS, Hospital Anxiety and Depression Scale; RSCS, Revised Scale for Caregiving; BDI-II, Beck Depression Inventory; NHP, Nottingham Health Profile; NPI, Neuropsychiatric Inventory; Zung, Zung Depression Self Rating Scale; EQ5D, EuroQoL.
